# iCDI-W2vCom: Identifying the Ion Channel–Drug Interaction in Cellular Networking Based on word2vec and node2vec

**DOI:** 10.3389/fgene.2021.738274

**Published:** 2021-09-09

**Authors:** Jie Zheng, Xuan Xiao, Wang-Ren Qiu

**Affiliations:** Department of Computer Engineering, Jingdezhen Ceramic Institute, Jingdezhen, China

**Keywords:** ion channels, word2vec, node2vec, data augmentation, LightGBM

## Abstract

Ion channels are the second largest drug target family. Ion channel dysfunction may lead to a number of diseases such as Alzheimer’s disease, epilepsy, cephalagra, and type II diabetes. In the research work for predicting ion channel–drug, computational approaches are effective and efficient compared with the costly, labor-intensive, and time-consuming experimental methods. Most of the existing methods can only be used to deal with the ion channels of knowing 3D structures; however, the 3D structures of most ion channels are still unknown. Many predictors based on protein sequence were developed to address the challenge, while most of their results need to be improved, or predicting web servers are missing. In this paper, a sequence-based classifier, called “iCDI-W2vCom,” was developed to identify the interactions between ion channels and drugs. In the predictor, the drug compound was formulated by SMILES-word2vec, FP2-word2vec, SMILES-node2vec, and ECFPs *via* a 1184D vector, ion channel was represented by the word2vec *via* a 64D vector, and the prediction engine was operated by the LightGBM classifier. The accuracy and AUC achieved by iCDI-W2vCom *via* the fivefold cross validation were 91.95% and 0.9703, which outperformed other existing predictors in this area. A user-friendly web server for iCDI-W2vCom was established at http://www.jci-bioinfo.cn/icdiw2v. The proposed method may also be a potential method for predicting target–drug interaction.

## Introduction

Ion channels are pore-forming membrane proteins that mediate the transport of ions in all living cells (Green, 1999) by controlling cell signaling during the change of the cellular physiology in organs (Gabashvili et al., 2007). For example, ion channels regulate the membrane potential by mediating the permeation of specific ion species through their transmembrane pores (Sumino et al., 2019). On other hand, dysfunction of ion channels may lead to over 55 different channelopathies (Qiang et al., 2018), such as epilepsy, arrhythmia, and type II diabetes (Tinaquero et al., 2020). It is also believed that the majority of patients with thyroid diseases and cardiac arrhythmia are ion channel blockers (Roepke et al., 2009) such that ion channels become important therapeutic targets.

As an essential step of drug discovery procedure, the identification of ion channel–drug interaction has lately become a hot topic issue since it involves costly, time-consuming, and challenging work during the development of new medicine (Knowles and Gromo, 2003). It has been reported that ionotropic glutamate receptor subfamily core is formed by two transmembrane helices and an intracellular reentrant pore helix (Amin et al., 2018); voltage-gated ion channels, including potassium channels and calcium channels, consist of six transmembrane helices (Kaufmann et al., 2019). Therefore, ion channels may be analyzed by using conventional methods of protein, and identification of ion channel–drug interactions inherently is a protein–drug interaction problem. There are many unpaired small molecule compounds for finding potentially new medications; many state-of-the-art computational methods have been developed to discover new drugs in the past few years (Xiao et al., 2013; Chen et al., 2018; Wang et al., 2020). Yamanishi et al. (2008) used statistical approach to predict the interaction between drugs and four targets on the base of the similarity. Wang et al. (2020) proposed a sequence-based method for identifying the protein–drug interaction. Since ion channel–drug interaction involves two substances, the methods that combine the biological information of ion channels and the chemical information of drugs are often used, and proper representation of proteins and drugs is essential to identify ion channel–drug with high efficiency.

For the feature extraction from protein, there are many classic algorithms applied to extract the feature from amino acid sequence, such as one hot encoding (Wang et al., 2019), composition of k-spaced amino acid pairs (CKSAAP; Chen et al., 2006), amino acid composition (AAC; Reczko and Bohr, 1994), and pseudo amino acid composition (PseAAC; Chou, 2005). The technology of natural language processing (NLP) was used to deal with secondary-structure prediction and subcellular localization (Elnaggar et al., 2020) in proteomics area. Furthermore, the deep learning techniques have been used to extract sequence features for protein–drug interaction. In order to get a protein feature (Wang et al., 2020), protein sequences were encoded using one hot encoding, and the information is then fed into a deep learning model, such as recurrent neural network (RNN), long short-term memory (LSTM), and gated recurrent unit (GRU; Shen et al., 2020).

For the processing of drug molecules, a variety of descriptors are used to represent drugs to fill the gap in analyzing the 3D structure for drugs, such as two-dimensional molecule graph (Bemis and Kuntz, 1992), MOL file (Qiu et al., 2020), simplified molecular-input line-entry system (SMILES; Jaeger et al., 2017), fingerprint and global descriptions of molecular by biophysical and chemical properties including the molecular weight (MW) and the partition coefficient for lipophilicity (Clark et al., 2019; Daina and Zoete, 2019). In these descriptors, drug molecules are usually represented with SMILES or Morgan fingerprint (Morgan FPs). The representation of SMILES string involves four overall steps: graph mol structure normalization, canonical labeling, tree traversal, and SMILES generation (O’Boyle, 2012), which is usually the start step for many computational methods such as recurrent neural networks (RNNs; Karimi et al., 2019), convolutional neural networks (CNNs; Huang et al., 2020), and graph neural networks (GNN; Tsubaki et al., 2019). Take extended connectivity fingerprints (ECFPs) as an example for Morgan FPs; all substructures around all heavy atoms of a molecule within a defined radius are generated and assigned to a unique identifier (called Morgan identifier), which would be compressed into a shorter fixed-length string (Zhou et al., 2020). The drug’s MOL file or SMILES can be acquired from http://www.kegg.jp/kegg/ or https://www.ebi.ac.uk/chembl/, and the software called OpenBabel^1^ could be used to convert the MOL file or SMILES into molecular fingerprint files in multiple formats: FP2, FP3, FP4, and MACSS.

Some novel encoding techniques were provided for proteins and drugs based on word2vec algorithm. As word2vec could map a class X of objects into a latent vector space where the geometric relationship is characterized by the semantic relationship between the objects (Grohe, 2020), it has been adapted to classify the protein sequences of protein families and predict the localization of proteins and the compound properties of drugs (Jaeger et al., 2017; Yang et al., 2018). Jaeger et al. (2018) proposed that the word2vec may identify the interaction between drugs and target proteins based on the amino acid sequences of proteins and the Morgan fingerprints of drugs. Zhang et al. (2019) further proposed a new predictor by using the amino acid sequences of proteins and the SMILES strings of drugs.

The study of ion channel--drug interaction networks is an important topic for drug development, while the computational prediction accuracies cannot meet the practical needs. Although deep learning methods are widely used in protein-target prediction, it is still in the exploratory stage for identifying ion channel--drug interaction. In addition, many research focused on constructing a complex neural network to extract interaction information, but for a method to encode a sequence, which is a crucial point of protein and drug representation, it gets rare attention. Thus, this paper was initiated in an attempt to develop a new powerful predictor based on the sequences of ion channels and the SMILES of drugs. There are four innovative characteristics of this work: (1) To get a better representation of protein, amino acid sequences were divided into words (k-grams) and encoded with the AAindex, which would be fed into word2vec to get distributed representations vectors of words. (2) To find the best way for the representation, two major descriptions, SMILES (SMILES_word2vec), and FP2 (FP2_word2vec), were separately tested for comparison on the basis of several combined features. (3) To augment the training dataset and get more information about the linking between different functional groups, the RDKit^2^, an open source chemistry informatics and machine learning toolkit, was used to generate different SMILES strings for the same molecule, and finally note2vec was applied to generate drug vectors (SMILES_node2vec). (4) To make full use of the drug and protein features mentioned above, the feature combination was performed deeply, and the prediction results improved significantly.

## Materials and Methods

### Benchmark Dataset

As more and more interactive pieces of information are in the database, such as DrugBank, KEGG, STITCH, ChEMBL, and TTD, many deep research studies have been carried out in drug discovery. In this work, the identification of ion channel–drug interaction is defined as a supervised prediction task in which a pair of counterparts interact with each other in the drug–target networks. The established KEGG database is utilized to define the pair of counterparts as it has an amount of interaction information of drugs and drug targets.

In this work, the benchmark dataset S is defined by:

(1) $$S=S^{+}\,\cup S^{-}$$

where S^+^ is the set of interactive ion channel–drug pairs, and S^–^ is the set of non-interactive ion channel–drug pairs, and the symbol ∪ represents the union in the set theory. The positive subset S^+^ contains 1,476 ion channel–drug pairs collected by Yamanishi et al. (2008).

To build the negative dataset, the approach was performed with the following steps: (i) Each pair in subset S^+^ was separated (drug ID and ion channel ID) into a single ion channel and drug. (ii) Each of the single ion channels was re-coupled with each of the single drug; therefore, the drug and ion channel are put into synthesized pairs in such a way. Those pairs that were in S^+^ were removed, and it was made sure that none of the pairs that were in S^+^ appeared in S^–^. (iii) The synthesized pairs were randomly picked until the number of selected pairs was the same as the number of pairs in S^+^. The dataset S^–^ contains 1,476 non-interactive ion channel–drug pairs.

An independent validation test is applied to evaluate the developed predictor for avoiding the overfitting of data from the reference (Yamanishi et al., 2008). The validation dataset, denoted as Check808, contains 404 interactive pairs and 404 non-interactive pairs. These pairs consist of the ion channels in S and new drug targets taken from the KEGG database. Any pairs have to be removed from the validation dataset if they appeared in the benchmark dataset.

Nuclear receptors (NRs) are another frequent target for drug development, but drug–NR pairs are more difficult in the protein–drug predict task. The dataset of NRs is used to verify the feature extraction method and the robustness of iCDI-W2vCom. The NR dataset contains a positive subset of 86 interactive drug–NR pairs, taken from the reference (Yamanishi et al., 2008) and a negative subset of 86 non-interactive pairs. The non-interactive pairs are different from the interactive pairs.

### Measurement

In the experiment, the performances of the predictor were evaluated with the following four metrics: accuracy (Acc), sensitivity (Sn), precision (Prec), and Matthews correlation coefficient (MCC; Jiao and Du, 2016). They were applied to evaluate the models and are shown in formula (2).

(2) $$\left\{ \begin{array}{ccc} A\,c\,c & = & \frac{T\,P+T\,N}{T\,P+T\,N+F\,P+F\,N}\\ S\,e\,n & = & \frac{T\,P}{T\,P+F\,N}\\ P\,r\,e\,c & = & \frac{T\,P}{T\,P+F\,P}\\ M\,C\,C & = & \frac{T\,P\times T\,N-F\,P\times F\,N}{\sqrt{\left(T\,P+F\,P\right)\,\left(T\,P+F\,N\right)+\left(T\,N+F\,P\right)\,\left(T\,N+F\,N\right)}} \end{array}\right.$$

### Representation of Ion Channel

The ion channel with the sequence length *l* is formulated in the following format:

(3) $$G=R_{1}\,R_{2}\,R_{3}\,R_{4}\,R_{5}\,R_{6}\,\ldots \,R_{3\,j+1}\,R_{3\,j+2}\,R_{3\,j+3}\,\ldots \,R_{\text{l}}$$

where *R*_1_ represents the first residue in ion channel sequence, *R*_2_ represents the second, …, and *R*_*l*_ represents the *l*-th one. How can we extract sequence information to represent an ion channel? We should translate a protein sequence into a digital vector that can well represent an ion channel.

In the article, three amino acids are divided into one word to construct the wordbook. As shown in the following example, a sequence of nine amino acids can be divided into three sets of non-overlapping 3-gram. Then *G* would be grouped as:

(4) $$G=\left(R_{1}\,R_{2}\,R_{3}\right)\,\left(R_{4}\,R_{5}\,R_{6}\right)\,\ldots \,\left(R_{3\,j+1}\,R_{3\,j+2}\,R_{3\,j+3}\right)$$

where *G* = *G*_1_ *G*_2_ …*G*_*j*_…*G*_*L*_ *G_L+1_*, *L = [l/3]*, *L* is a round down of *l*/3, and *G_L+1_* may be *Ø* or only contains one or two residues, which are due to the remainder of *l*/3.

The AAindex database indexes are the biophysical and chemical properties of amino acids and pairs of amino acids^3^ (Zhou et al., 2020). In this paper, five groups of AAindex were selected for the experiment, which are the same as the reference (Wang et al., 2020), and the corresponding values of amino acids are shown in Table 1. The AAindex1 physicochemical property stands for “hydropathy index,” AAindex2 for “molecular weight,” AAindex3 for “isoelectric point (PI),” AAindex4 for “pK-N,” and AAindex5 for “pK-C.”

**TABLE 1:** Five physicochemical property codes for each of the 20 native amino acids.

**Amino acid**	**Five physicochemical property codes**				
	**AAindex1**	**AAindex2**	**AAindex3**	**AAindex4**	**AAindex5**
A	1.8	89.09	6.00	9.69	2.34
C	2.5	121.15	5.05	8.35	1.92
D	–3.5	133.10	2.77	9.60	1.88
E	–3.5	147.13	3.22	9.67	2.10
F	2.8	165.19	5.48	9.18	2.16
G	–0.4	75.07	5.97	9.78	2.35
H	–3.2	155.16	7.59	9.17	1.82
I	4.5	131.17	6.02	9.68	2.36
K	–3.9	146.19	9.74	9.18	2.16
L	3.8	131.17	5.98	9.60	2.36
M	1.9	149.21	5.74	9.21	2.28
N	–3.5	132.12	5.41	8.80	2.02
P	–1.6	115.13	6.30	10.64	1.95
Q	–3.5	146.15	5.65	9.13	2.17
R	–4.5	174.20	10.76	8.99	1.82
S	–0.8	105.09	5.68	9.21	2.19
T	–0.7	119.12	5.66	9.10	2.09
V	4.2	117.15	5.96	9.62	2.32
W	–0.9	204.24	5.89	9.44	2.43
Y	–1.3	181.19	5.66	9.11	2.20

With the AAindex values, the ion channel sequence *G* would be encoded into a vector shown as follows:

(5) $$G=\left(g_{1}\,g_{2}\,\ldots \,g_{\text{j}}\,\ldots \,g_{\text{L}}\,g_{L+1}\right),g_{\text{j}}=\frac{\sum_{R\in G_{\text{j}}}\rho \,\left(R\right)}{∥G_{\text{j}}∥}$$

where *ρ(R)* is the AAindex value of reside *R*, and ||*G*_*j*_|| is the number of residues in group *G*_*j*_, *j = 1,2,…,L*(*L+1* when the remainder of *l/3* is not equal to zero). Therefore, a new corpus of words is constructed through AAindex indices. The corpus may reduce the number of words made of amino acids string. For example, a word made of AAindex indices may take the place of a triplet composed of amino acids D, E, and F with a total of nine words. Such an expression may also combine triplicates with similar properties together. In particular, the hydrophilic coefficient is used to code the triplicates; the words “EFG” and “DFG” will be combined into the same word.

Although word2vec is an unsupervised method, here, an auxiliary prediction task was defined to train the word representation model with one of the following two approaches: (1) continuous bag-of-words (CBOW), which may predict a word from the context words, and (2) Skip-gram, which predicted the context based on a word. In CBOW, the order of words in the context is not important due to the bag-of-words assumption, while the adjacent words are assigned with higher weights in Skip-gram. We mainly used the Skip-gram model to train the word2vec model.

The classical Skip-gram model consists of an input layer, projection layer, and output layer. The model learns information from corpus and stores the derived knowledge in weights θ. The positive samples of Skip-gram model are words *g*_*I*_ and their contexts *C(g_*I*_)*. Contexts of a word *g*_*I*_, which was derived from a window of size k around the word: *C(g)* = *g_i–k_*,…,g_*i–1*_,g_*i*__+__1_,…,g_*i+k*_, where the window size k is a parameter for word2vec; the negative samples are generated by relatively simple method called negative sampling.

**FIGURE 1:**
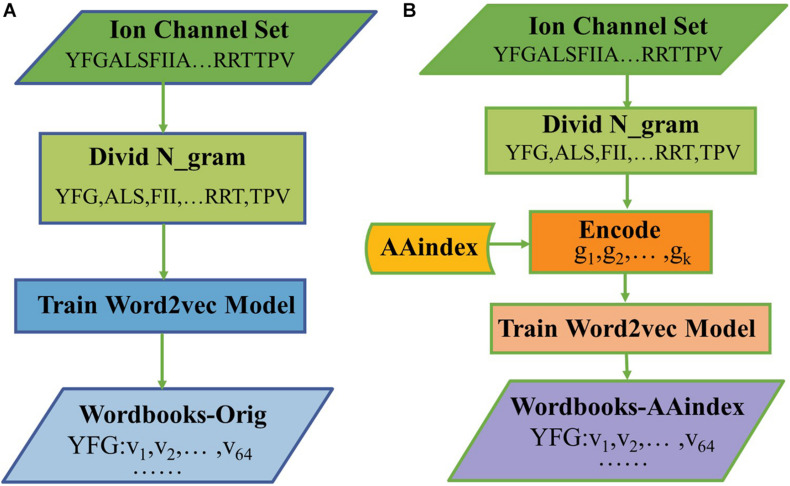
The processes of constructed **(A)** Wordbooks-Orig and **(B)** Wordbooks-AAindex.

The hyperparameters of Skip-gram were set as follows: the embedding dimension is *d* = 64, the context window size is *k* = 4, and the number of negative examples is *k* = 8. After training for 30 epochs, we get a final wordbook. The process of constructing the original triplicate workbooks (Wordbooks-Orig) is shown in Figure 1A, as a result, each word would be represented with a 64-dimensional vector, and each word and its corresponding vector are storied in “Wordbooks-Orig.” The process of constructing a Wordbooks-AAindex is shown in Figure 1B. Finally, each word encoded with AAindex indices is represented with a 64-dimensional vector; the words and their vectors are storied in “Wordbooks-AAindex.”

**FIGURE 2:**
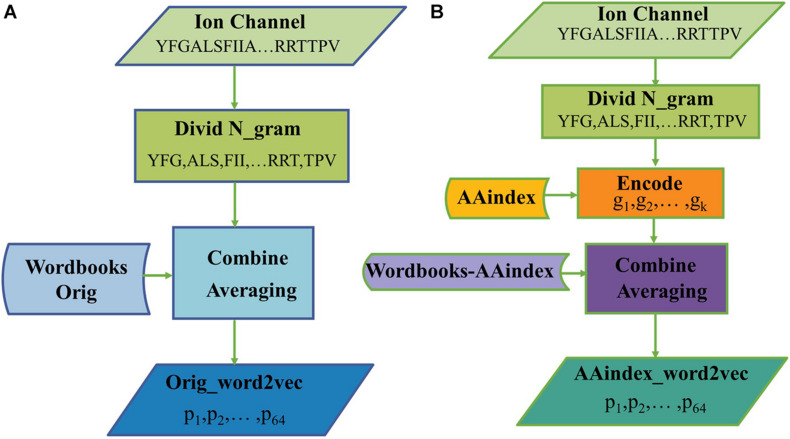
The processes of generating protein representation through **(A)** Orig_word2vec and **(B)** AAindex_word2vec.

Figure 2 illustrates the process of generating protein representation through Orig_word2vec and AAindex_word2vec, respectively. In Figure 2A, amino acid sequences were divided into 3-gram, and then looked up the “Wordbooks-Orig” to obtain a vector for every word. The representation for a protein is finally obtained by averaging every word over the length dimension of the protein. In Figure 2B, every word was encoded in AAindex_word2vec with AAindex indices, which may help generate more efficient vectors of words. For example, when to handle a ion channel sequence through AAindex_word2vec, we divide it into 3-gram and encode every word with AAindex indices, then the “Wordbooks-AAindex” were looked up to obtain a vector for every word. The representation for a protein is finally obtained by averaging every word over the length dimension of the protein.

**FIGURE 3:**
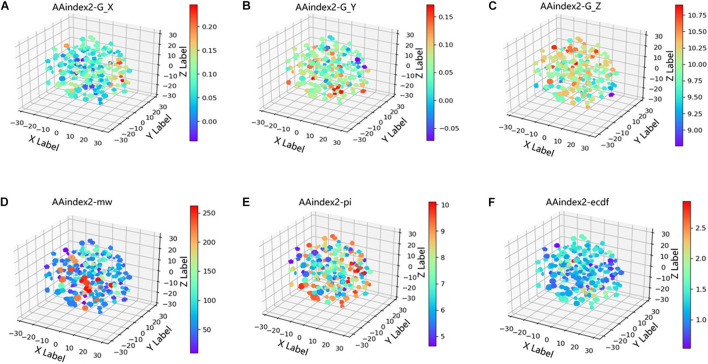
Projected the AAindex2_word2vec from 64-dimensional to 3D space. **(A–C)** The coordinates of points are the values after dimensionality reduction, and the color is three coefficients derived from the second order gray model. **(D–F)** The coordinates of points are the values after dimensionality reduction, and the color is isoelectric point(PI), molecular weight (MW), extinction coefficient of different segments (ECDF).

In the word2vec model, the features learned at each layer are not visual. To explore what information word vectors imply, t-distributed stochastic neighbor embedding (t-SNE; van der Maaten and Hinton, 2008), a non-linear dimensionality reduction algorithm, was used to projected the vectors of ion channels from 64-dimensional to 3D space. As shown in Figure 3, the coordinates of points are the values after dimensionality reduction, and the color of points are termed G_X, G_Y, and G_Z, respectively, which are three coefficients derived from the second-order gray model (Xiao et al., 2008). In Figures 3A–C, the coordinates of points are the values after dimensionality reduction, and the color is three coefficients derived from the second-order gray model. In Figures 3D–F, the coordinates of points are the values after dimensionality reduction, and the color is the PI, MW, and extinction coefficient of different segments (ECDF). We can find that word vectors can learn implicitly the three coefficients derived from the second-order gray model (G_X, G_Y, and G_Z), PI, MW, and ECDF.

### Representation of Drug

Due to the complex three-dimensional structure and unique properties of drugs, the characterization of drug performance stored in the computer often lose a lot of information. Fortunately, there are many approaches to represent drugs with different characteristics, which involve molecular diagram, Morgan FPs, SMILES, and so on.

(1) Representing drug with word2vec

**FIGURE 4:**
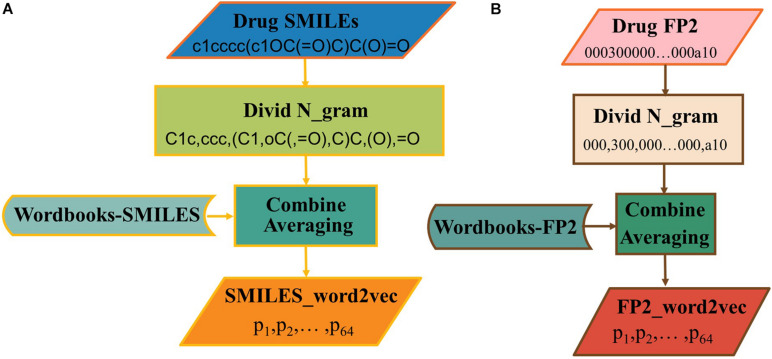
The processes of generating **(A)** SMILES_word2vec and **(B)** FP2_word2vec.

The word2vec has been used to generate vectors *via* SMILES or FP2. There is still a need to know which one is the best choice for this issue. As shown in Figure 4A, the SMILES string can be divided into n-gram. Here, the sequence of the drug was divided into non-overlapping 3-gram, and word2vec algorithm was selected to generate the word vector. In addition, the process to construct FP2_word2vec is shown in Figure 4B.

(2) Representing drug with node2vec

The node2vec (Grover and Leskovec, 2016) may also represent the drug feature, and it captures the information between nodes in networks (Grohe, 2020; Shen et al., 2021). Here, the node2vec is applied to obtain node features for a complementary characteristic of the drug.

Simplified molecular-input line-entry system strings are divided into functional groups taken as the nodes of the network. The functional group comprises multiple atoms or atomic groups, and its physicochemical properties are fundamental in the pharmacodynamic phase of the mechanisms of action of many drugs (Silva et al., 2019). In the SMILES, the SMILES strings are marked: no mark for single key, ‘‘ = ’’ for double key, ‘‘#’’ for triple key, and ‘‘(‘‘ or ‘’)’’ for branch chain^4^. The SMILES of the drug molecules is separated by the special marks, and every part is taken as a node.

**FIGURE 5:**
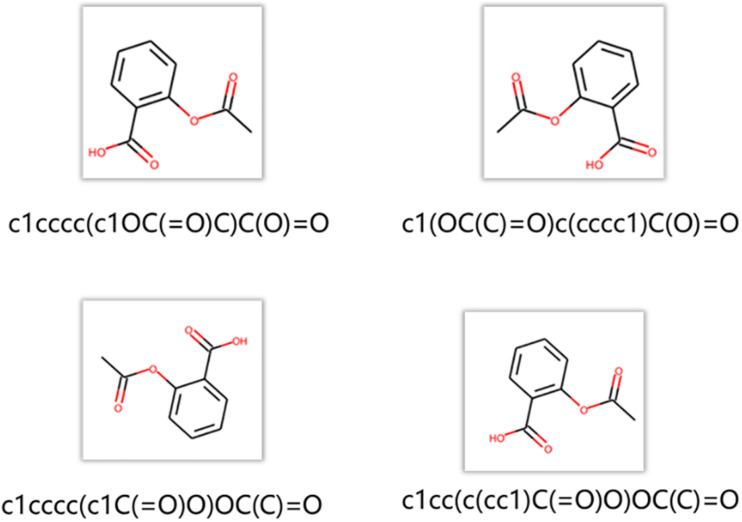
Generation of different SMILES strings for the same molecule.

The node2vec regards a random path generated by a random walk as a set of words. A data augmentation approach is chosen to generate more paths and get more information between nodes in the networks. As shown in Figure 5, RDKit was used to generate different SMILES strings for the same molecule. These SMILES strings are all valid structures. RDKit generates different SMILES strings by rotating the molecular graph to generate different SMILES strings whose starting atom and the direction of graph enumeration are randomly selected. In the procedure of training node2vec, data augmentation approach can better obtain the connection relation between functional groups and get a better node vector (Tetko et al., 2020).

**FIGURE 6:**
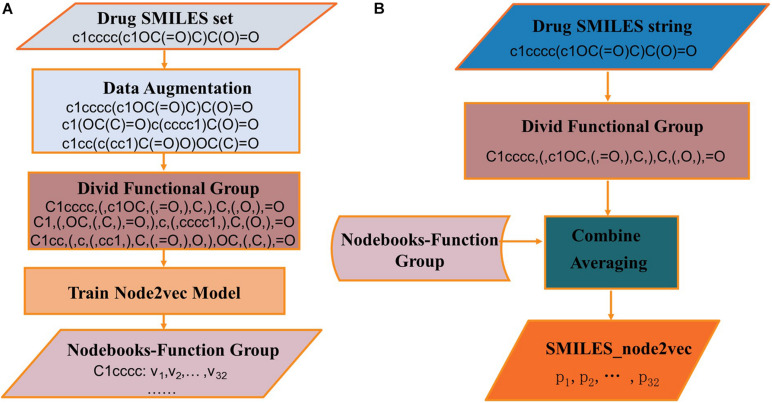
The processes of **(A)** constructing Wordbooks-Orig and **(B)** generating SMILES_node2vec.

The process of generating SMILES_node2vec is shown in Figure 6. In Figure 6A, the SMILES strings are divided into function groups, and the Nodebooks-Function Group is generated by the node2vec model. In Figure 6B, SMILES strings were first divided into function groups, and then the “Nodebooks-Function Group” was looked up to obtain a vector for every word. The representation for a drug is finally obtained by averaging every word over the length dimension of the drug.

(3) Representing drug with ECFPs

In the drug database, the drugs in SMILES format (Weininger, 1988) may be further fed into RDKit, to extract their ECFPs (Zhou et al., 2020), such that a drug can be represented by a 1,024-D binary vector.

The multiple ways of representing a molecule and the different levels of uncertainty regarding those representations have been a central part of this expertise. In this paper, we try to improve the accuracy of ion channel–drug interaction by feature combination. In Rayhan et al. (2019), it is shown that an ensemble boosting method performs much better than other methods in DTI prediction. The feature subset of drugs includes SMILES-word2vec (Feature SMILES), FP2-word2vec (Feature FP2), SMILES-node2vec (Feature Node), and ECFP (Feature ECFP). As shown in Table 2, the subsets of the feature mentioned above are tested with the LightGBM classifier *via* fivefold cross-validation, and the feature dimension of the six learners are shown in the last column of the table.

**TABLE 2:** Profile of the six learners.

	**AAindex1_word2vec**	**SMILES-word2vec**	**FP2-word2vec**	**SMILES-node2vec**	**ECFP**	**Number of features**
Learn-1	√	√				128
Learn-2	√		√			128
Learn-3	√	√		√		160
Learn-4	√		√	√		160
Learn-5	√	√	√	√		224
Learn-6	√	√	√	√	√	1,248

*ECFP, extended-connectivity fingerprints.*

### Prediction Engine

Once the protein and drug were represented by vectors, some machine learning models would be utilized for the prediction process. We compared the performance of different algorithms involved in LightGBM (LGB; Ke et al., 2017), gradient boosting decision tree (GBDT; Friedman, 2000), random forest (RF; Liaw and Wiener, 2002), and deep neural networks (DNNs) on the ion channel dataset (Pedregosa et al., 2011). All these models were implemented in Python 3 (Python ≥3.6) environment with LightGBM package (Zhang et al., 2017) and Scikit-learn library (Pedregosa et al., 2011).

## Results and Discussion

The original triplicates workbooks (Orig_word2vec) were used for the first experiment, and five AAindex_word2vec were used for comparison. Results are listed in Table 3. It was found that the AAindex_word2vec for the proteins improved the performance of the classifier greatly. As listed in the tables, bold values mean that they are the best scores compared with other methods.

**TABLE 3:** Performance of different protein representations on the ion channel dataset.

**Protein feature**	**AUC**	**Acc (%)**	**Prec (%)**	**Sen (%)**	**MCC**
Orig_word2vec	0.9720	91.29	90.75	91.91	0.8262
AAindex1_word2vec	0.9703	91.95	**91.18**	92.95	0.8402
AAindex2_word2vec	0.9717	91.69	90.76	92.72	0.8344
AAindex3_word2vec	0.9700	91.77	91.15	92.41	0.8355
AAindex4_word2vec	0.9676	91.22	90.85	91.65	0.8247
AAindex5_word2vec	0.9682	91.22	90.32	92.20	0.8248
All_word2vec	**0.9730**	**92.13**	91.17	**93.13**	**0.8430**

*ACC, accuracy; Prec, precision; Sen, sensitivity; MCC, Matthews correlation coefficient. Bold values mean that they are the best scores compared with other methods.*

Comparing the experimental results of original drug expression PF2, the SMILES string as input, and using word2vec to extract features in other articles, this work combined the features of drugs in different descriptions. We can find that the combination of SMILES_word2vec (Feature SMILES), SMILES_node2vec (Feature node), FP2_word2vec (Feature FP2), and ECFP (Feature ECFP) has achieved the optimal effect. Table 4 shows the result comparison of fivefold cross-validation. The descriptors of molecules mentioned above are ambiguous or missing some information, but those descriptors are highly complementary, and experimental findings show that drug feature combination is useful.

**TABLE 4:** Performances of different drug descriptions on the ion channel dataset.

	**AUC**	**Acc (%)**	**Prec (%)**	**Sen (%)**	**MCC**
Learner-1	0.9598	90.24	88.98	91.61	0.8054
Learner-2	0.9675	91.26	90.26	92.33	0.8256
Learner-3	0.9625	91.33	90.41	92.36	0.8271
Learner-4	0.9703	91.25	90.16	92.46	0.8257
Learner-5	0.9696	91.51	90.16	**93.03**	0.8310
Learner-6	**0.9703**	**91.95**	**91.18**	92.95	**0.8402**

*Bold values mean that they are the best scores compared with other methods.*

**FIGURE 7:**
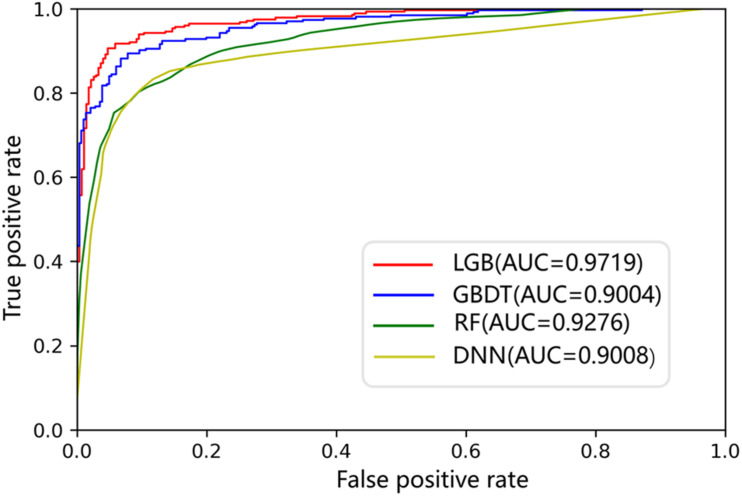
ROCs of different models on the channel–drug interaction dataset.

**FIGURE 8:**
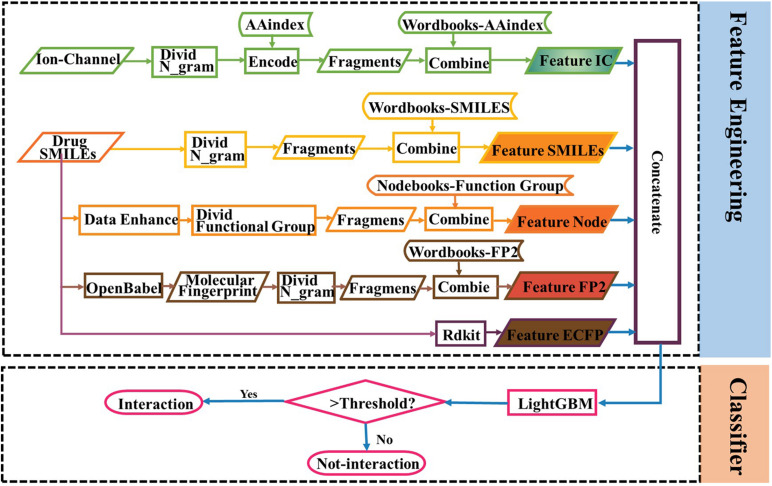
Flowchart of the iCDI-W2vCom model.

The AUC curves of LGB, GBDT, RF, and DNN on the ion channel dataset are shown in Figure 7. The LightGBM approach performs quite high AUCs in the test such that it is selected as the predictor. The parameter values of LightGBM model are num_leaves of 48, max_depth of 9, learning_rate of 0.03, n_estimators of 600, min_child_samples of 3, and other parameters are set with their default values. The flowchart of the proposed iCDI-W2vCom model is shown in Figure 8. The model inputs the SMILES strings of accessible drugs and the amino acid sequences of ion channels. The feature subsets are fed into the LightGBM predictor for a final prediction with a fivefold cross-validation method.

Predictor was optimized by using AAindex1_word2vec for ion channel and feature combination for drug. Table 5 shows the results of the proposed model on the ion channel–drug interaction dataset *via* fivefold cross-validation. The ICDI-W2vCom based on both the word2vec model and node2vec model has an average AUC of 0.9703, Acc of 91.95%, precision of 91.18%, sensitivity of 92.95%, MCC of 0.8402 vs. other newly publish methods of 0.8900, 89.10%, 88.30%, 91.20%, 0.8060, respectively. Thus, our performance has been improved, surpassing other existing classifiers as shown in Table 6.

**TABLE 5:** Results of the proposed model in channel–drug interaction dataset.

**Test**	**AUC**	**Acc (%)**	**Prec (%)**	**Sen (%)**	**MCC**
1	0.9680	90.89	89.98	91.82	0.8180
2	0.9650	90.53	87.50	94.07	0.8132
3	0.9779	92.70	92.73	93.07	0.8579
4	0.9695	93.08	93.04	93.12	0.8615
5	0.9711	92.53	92.65	92.42	0.8506
**Average**	0.9703 ± 0.0043	91.95 ± 1.03	91.18 ± 2.15	92.95 ± 0.73	0.8402 ± 0.0205

**TABLE 6:** Performances of different methods on channel–drug interaction dataset.

	**RFDT** (Wang et al., 2018)	**Wang** (Wang et al., 2020)	**The proposed method**
AUC	0.8900 ± 0.0200	0.8895 ± 0.0146	**0.9703**±**0.0043**
Acc (%)	89.10 ± 1.50	88.82 ± 0.65	**91.95**±**1.03**
Prec (%)	87.60 ± 1.60	88.30 ± 0.16	**91.18**±**2.15**
Sen (%)	91.20 ± 1.90	89.50 ± 0.73	**92.95**±**0.73**
MCC	0.8060 ± 0.024	0.7763 ± 0.0134	**0.8402**±**0.0205**

*Bold values mean that they are the best scores compared with other methods.*

Using the ion channel–drug interaction benchmark datasets as training dataset and Check808 as an independent test set, different algorithms were tested, and the results are listed in Table 7.

**TABLE 7:** Performance comparisons on Check808.

	**RF**	**DNN**	**GBDT**	**LGB**
AUC	0.8916	0.9058	0.9370	**0**.**9630**
Acc (%)	81.68	85.64	87.25	**90**.**47**
Prec (%)	86.23	82.22	86.16	**89**.**64**
Sen (%)	78.10	89.44	88.43	**91**.**35**
MCC	0.6388	0.7170	0.7454	**0**.**8096**

*RF, random forest; DNN, deep neural network; GBDT, gradient boosting decision tree; LGB, LightGBM. Bold values mean that they are the best scores compared with other methods.*

According to the results, the features we generate can very well characterize the channel–drug interactions, and the default classifier LGB has a better generation ability by comparison with RF, DNN, and GBDT.

The proposed method achieved good performance in the NR dataset. The results are listed in Table 8. Compared with the previously published articles (Wang et al., 2018; Wang et al., 2020), the ICDI-W2vCom has great AUC, Acc, Sen, and MCC values such that it gets good robustness.

**TABLE 8:** Performances of different methods on NR–drug interaction datasets.

	**RFDT** (Wang et al., 2018)	**Lei** (Wang et al., 2020)	**The proposed method**
AUC	0.7230 ± 0.0380	0.8074 ± 0.0933	**0.9014**±**0.0325**
Acc (%)	71.10 ± 4.60	82.22 ± 3.17	**87.14**±**3.23**
Prec (%)	68.00 ± 12.10	**84.74**±**12.53**	84.73 ± 4.42
Sen (%)	75.90 ± 10.00	79.98 ± 12.70	**83.66**±**4.55**
MCC	0.5790 ± 0.0400	0.6573 ± 0.0699	**0.7338**±**0.0673**

*Bold values mean that they are the best scores compared with other methods.*

## Conclusion

In the research, the proposed model based on AAindex encoding sequences and word2vec algorithm significantly improved the learning ability of predictors. This inspires us that, in small datasets, coding protein words according to their physical and chemical properties may reduce the number of words in the lexicon, which trained the word2vec model faster and generate a high quality of word. By using conventional protein processing methods and knowledge, the parameters of deep learning could be reduced, and the computation would be simplified. Furthermore, by using the t-SNE algorithm to project the vectors of ion channels from 64-dimensional to 3D space vectors, vectors can learn implicitly features represented by other protein-encoding methods (for example, the gray model) and physicochemical properties. This work suggests that word2vec can also be accepted in ML as many previous works do.

The multiple ways of representing a molecule and the different levels of uncertainty regarding those representations have been a central part of this expertise. Therefore, we try to fuse drug information of different descriptions to represent drugs comprehensively. In this paper, the expression of drugs was enhanced through the combination of different features, and the performance of the classifier was improved greatly.

## Data Availability Statement

The original contributions presented in the study are included in the article/supplementary material, further inquiries can be directed to the corresponding authors.

## Author Contributions

XX conceived and designed the experiments. JZ performed the extraction of features, model construction, model training, and evaluation, and drafted the manuscript. XX and W-RQ supervised the project and revised the manuscript. All authors read and approved the final manuscript.

## Conflict of Interest

The authors declare that the research was conducted in the absence of any commercial or financial relationships that could be construed as a potential conflict of interest.

## Publisher’s Note

All claims expressed in this article are solely those of the authors and do not necessarily represent those of their affiliated organizations, or those of the publisher, the editors and the reviewers. Any product that may be evaluated in this article, or claim that may be made by its manufacturer, is not guaranteed or endorsed by the publisher.

## Footnotes

1
http://openbabel.org/

2
http://www.rdkit.org/

3
http://www.genome.jp/aaindex/

4
https://www.daylight.com/meetings/summerschool98/course/dave/smiles-bonds.html
